# Supplementary material for: COVID-19 Vaccine Does Not Increase the Risk of Disease Flare-Ups among Patients with Autoimmune and Immune-Mediated Diseases
Source: J Pers Med. 2021 Dec 2;11(12):1283. doi: 10.3390/jpm11121283 (PMC8707188; doi:10.3390/jpm11121283)
Supplement: Supplementary file 1 [file jpm-11-01283-s001.zip › jpm-1453160-supplementary.pdf]

Journal of Personalized Medicine

MDPI

Supplementary Materials

Disease activity evaluation chart

Q1: Did you experience a worsening of the symptoms related to your immune disease?

Table S1. Clinical and biological features for each immune disease.

	Clinical evaluation	Biological features
Rheumatoid arthritis [1]	joint pain, joint swelling, stiffness and constitutional symptoms	inflammatory syndrome
Systemic lupus erythematosus [2]	fever, pericarditis, pleurisy, mucosal ulcers, alopecia, new rash, arthritis, proximal muscle aching/weakness, ulcerations, gangrene, recent cerebrovascular accident, severe persistent headache, visual disturbance, altered mental function, psychosis, seizure	increased ESR[1], low complement, increased anti-dsDNA[2], thrombocytopenia, leukopenia, proteinuria, urinary casts
Sjögren's syndrome/Sicca[3] [3]	dryness, limb pain (joint or muscular), mental fatigue	inflammatory syndrome
Ankylosing spondylitis [4]	neck, back or hip pain, joint pain or swelling, morning stiffness and fatigue	inflammatory syndrome
Psoriatic arthritis/psoriasis [5,6]	joint pain, joint swelling, tender joint and/or skin redness, thickness, desquamation	inflammatory syndrome, hyperuricemia
Systemic sclerosis/ limited scleroderma [7]	scleroderma, digital necrosis, arthritis	ESR, low complement
Antiphospholipid syndrome	vascular thrombosis, pregnancy morbidity	-
Systemic vasculitis [8]	myalgia, arthralgia/arthritis, fever, weight loss, cutaneous lesions (infarct, purpura, ulcer, gangrene) mucous or eyes lesions, cardiovascular (loss of pulses, valvular heart disease, pericarditis, ischaemic cardiac pain, cardiomyopathy, congestive heart failure), abdominal (peritonitis, bloody diarrhoea), renal (hypertension, proteinuria, haematuria), nervous system features	inflammatory syndrome, altered renal function
Other AIRD [4,5]	muscle weakness, skin manifestations, dysphagia	inflammatory syndrome, elevated serum levels of creatine kinase/lactat dehydrogenase, aspartate or alanine aminotransferase
Inflammatory bowel disease	abdominal pain, stool pattern, general well being, arthritis/arthralgia, uveitis, abdominal mass, weight change	anemia, inflammatory syndrome
Celiac disease	bloating, diarrhea	increasing titers of celiac disease -specific serology, worsening of nutritional parameters
Primary biliary cholangitis	fatigue, jaundice	increase in cholestatic enzymes, bilirubin levels or Immunoglobulin M
Autoimmune hepatitis	fatigue, jaundice	increase in liver function tests
Myasthenia gravis [9]	double vision, talking, swallowing, chewing, breathing, neck flexion or extension, shoulder or hip muscle weakness	-
Multiple sclerosis [10]	new neurological symptoms (ataxia, dysarthria, decrease in touch, bowel and bladder dysfunction, visual symptoms)	-
Hematological diseases [6]	pailness, purpura, petchiae	anemia, thrombocytopenia
Cutaneous diseases [7]	new skin lesions	inflammatory syndrome
Autoimmune thyroid disease	fatigue, constipation, alopecia, myalgia, arthralgia, muscle weakness	thyroid dysfunction
Other non-AIRD [8]	cough, joint pain, joint swelling, chills, fever	inflammatory syndrome, hyperglycemia

[1] ESR- erythrocyte sedimentation rate; [2] anti-dsDNA- anti-double stranded deoxyribonucleic acid; [3] Sicca- Sicca syndrome (xerostomia, xeroftalmia); [4] AIRD—autoimmune rheumatic diseases; [5] Other AIRD (autoimmune rheumatic diseases)-Dermatomyositis/polymyositis and Mixed connective tissue disease, [6] Hematological diseases- Hemolytic anemia, Paroxysmal Nocturnal Hemoglobinuria, Idiopathic thrombocytopenia, [7] Cutaneous diseases- vitiligo, cutaneous lupus, cutaneous vasculitis, pemphigus, [8] Other non AIRD- Sarcoidosis, Type 1 diabetes mellitus, Hyper IgD syndrome.

Questions for patients with clinical features suggestive for an increase in disease activity:

Q2: Did you require hospital admission for your symptoms? Did your treating physician established that you had a flare (including targeted medical evaluation for the immune disease)

Q3: Did you biological panel reveal inflammatory syndrome (increased ESR and/or CRP) and/or modified biological features suggestive for disease activity? (Table S1)

Q4: Did you require changes in the baseline treatment (increase in dose, frequency, or type of medication) in order to relieve your symptoms?

Flare was considered if the patient had typical clinical symptoms and/or at least one of the three additional arguments (Q2-Q4). We defined self-reported flare as any worsening of the disease reported by patients occurring in the time between visits.

References

1. Wells, G.; Becker, J.-C.; Teng, J.; Dougados, M.; Schiff, M.; Smolen, J.; Aletaha, D.; Van Riel, P.L.C.M. Validation of the 28-joint Disease Activity Score (DAS28) and European League Against Rheumatism response criteria based on C-reactive protein against disease progression in patients with rheumatoid arthritis, and comparison with the DAS28 based on erythrocyte sedimentation rate. *Ann. Rheum. Dis.* **2008**, *68*, 954–960, doi:10.1136/ard.2007.084459.
2. Arora, S.; Isenberg, D.A.; Castrejon, I. Measures of Adult Systemic Lupus Erythematosus: Disease Activity and Damage. *Arthritis Rheum.* **2020**, *72*, 27–46, doi:10.1002/acr.24221.
3. Seror, R.; Ravaud, P.; Mariette, X.; Bootsma, H.; Theander, E.; Hansen, A.; Ramos-Casals, M.; Doerner, T.; Bombardieri, S.; Hachulla, E.; et al. EULAR Sjögren's Syndrome Patient Reported Index (ESSPRI): development of a consensus patient index for primary Sjögren's syndrome. *Ann. Rheum. Dis.* **2011**, *70*, 968–972, doi:10.1136/ard.2010.143743.
4. Medscape Home Page. Available online: https://reference.medscape.com/calculator/299/basdai (accessed on 27 October 2021).
5. Louden, B.A.; Pearce, D.J.; Lang, W.; Feldman, S.R. A simplified psoriasis area severity index (SPASI) for rating psoriasis severity in clinic patients. *Dermatol. Online J.* **2004**, *10*, 7,, doi:10.5070/d318w9j736.
6. Schoels, M.M.; Aletaha, D.; Alasti, F.; Smolen, J.S. Disease activity in psoriatic arthritis (PsA): defining remission and treatment success using the DAPSA score. *Ann. Rheum. Dis.* **2015**, *75*, 811–818, doi:10.1136/annrheumdis-2015-207507.
7. Valentini, G.; Bencivelli, W.; Bombardieri, S.; D'Angelo, S.; Della Rossa, A.; Silman, A.J.; Black, C.M.; Czirjak, L.; Nielsen, H.; Vlachoyiannopoulos, P.G. European Scleroderma Study Group to define disease activity criteria for systemic sclerosis. III. Assessment of the construct validity of the preliminary activity criteria. *Ann. Rheum. Dis.* **2003**, *62*, 901–903, doi:10.1136/ard.62.9.901.
8. European Vasculitis Society Home Page. Available online: https://vasculitis.org/disease-scoring/ (accessed on 2 November 2021).
9. Burns, T.M.; Conaway, M.; Sanders, D.B. The MG Composite. *Neurol.* **2010**, *74*, 1434–1440, doi:10.1212/wnl.0b013e3181dc1b1e.
10. Kaunzner, U.W.; Al-Kawaz, M.; Gauthier, S.A. Defining Disease Activity and Response to Therapy in MS. *Curr. Treat. Options Neurol.* **2017**, *19*, 20, doi:10.1007/s11940-017-0454-5.
